# THE ROLE OF FAMILY CAREGIVING CONFIGURATIONS AND SUPPORTS IN OLDER ADULT OUTCOMES

**DOI:** 10.1093/geroni/igac059.1642

**Published:** 2022-12-20

**Authors:** Natalie Chong

**Affiliations:** Brandeis University, Waltham, Massachusetts, United States

## Abstract

This study uses data from the 2017-2018 National Health and Aging Trends Study and the 2017 National Study of Caregiving to examine whether caregiving network configuration factors (e.g., if and how multiple caregivers of the same care recipient distribute tasks, responsibility and disagreement among caregivers, involvement of paid caregivers) and caregiver supports (e.g., emotional support, skills training, respite services) are associated with consequences of unmet needs among older adults. Findings from this study will provide greater understanding of how caregiving contexts shape the quality of care and health of older adults who rely on help from family caregivers.

